# Optimal Dietary *Dunaliella salina* Supplementation Enhances Growth, Antioxidant Capacity, Immune Response, Stress Tolerance, and Carotenoid‐Based Pigmentation in Juvenile Red Swamp Crayfish (*Procambarus clarkii*)

**DOI:** 10.1155/anu/3727931

**Published:** 2026-02-07

**Authors:** Chen Qian, Jinghao Li, Yawen Zhang, Yongxu Cheng, Jiayao Li

**Affiliations:** ^1^ Key Laboratory of Integrated Rice-Fish Farming Ecosystem, Ministry of Agriculture and Rural Affairs, Shanghai Ocean University, Shanghai, 201306, China, shou.edu.cn; ^2^ Centre for Research on Environmental Ecology and Fish Nutrition of the Ministry of Agriculture and Rural Affairs, Shanghai Ocean University, Shanghai, 201306, China, shou.edu.cn; ^3^ National Demonstration Center for Experimental Fisheries Science Education, Shanghai Ocean University, Shanghai, 201306, China, shou.edu.cn; ^4^ Chinese Mitten Crab Industry Research Center of Jiangsu Province, Nanjing, 210000, China; ^5^ School of Marine Sciences, Ningbo University, Ningbo, 315832, China, nbu.edu.cn

**Keywords:** antioxidant capacity, carotenoid deposition, *Dunaliella salina*, immunity, nonspecific immunity, pigment deposition, *Procambarus clarkii*, stress tolerance

## Abstract

To meet the growing market demand for high‐quality red swamp crayfish (*Procambarus clarkii*), nutritional strategies are needed to improve both health and visual traits. The microalga *Dunaliella salina*, renowned for its rich natural β‐carotene content, presents a promising solution. This study investigated the effects of dietary supplementation with *D. salina* powder at five graded levels (0%, 0.34%, 0.67%, 1.34%, and 2.00%) on juvenile crayfish over a 60‐day feeding trial. Results demonstrated that weight gain rate (WGR) and specific growth rate (SGR) were highest in the 2.00% supplementation group. Notably, a low inclusion level of 0.34% significantly promoted ovarian development, as evidenced by the highest gonadosomatic index (GSI) and digestive enzyme activities. Antioxidant capacity (AOC, total superoxide dismutase [T‐SOD], total AOC [T‐AOC]) and nonspecific immunity (alkaline phosphatase [AKP]) were maximally enhanced at 0.67% inclusion. Most importantly, dietary *D. salina* was efficiently converted to astaxanthin and deposited in tissues, with carapaces and ovaries coloration parameters showing a strong, dose‐dependent correlation with carotenoid accumulation. Regression analysis identified an optimal inclusion range of 1.23%–1.53% for maximizing carotenoid deposition, immune function, and AOC. Furthermore, the 2.00% supplementation group exhibited the highest tolerance to air exposure stress. Our findings provide definitive, data‐driven insights for the precise application of *D. salina* in functional crayfish feeds, facilitating the industry’s transition from quantity‐focused production to quality‐ and value‐driven aquaculture.

## 1. Introduction

The red swamp crayfish (*Procambarus clarkii*) is one of the primary freshwater crustaceans in China, characterized by the highest yield and significant economic value [1]. However, as consumer expectations evolve, the market now demands higher standards in terms of product safety, nutritional value, and visual appeal, particularly a desirable reddish pigmentation. This shift is driving the industry from “quantity‐driven expansion” toward “quality and efficiency improvement” [2], thereby necessitating the development of novel, multifunctional feeds that not only support basic growth but also enhance health, improve product quality, and are sustainable and cost‐effective. Dietary supplementation of carotenoids represents a viable strategy for improving the physiological condition of premium red swamp crayfish, especially in terms of stress resistance and desirable commercial attributes during the farming process [3].

Carotenoid additives in aquatic feed have garnered widespread attention, such as astaxanthin, β‐carotene, canthaxanthin, lutein, and zeaxanthin. Compared to the more expensive astaxanthin, β‐carotene offers a more cost‐effective alternative. Numerous studies have demonstrated that the incorporation of β‐carotene into feed formulas of crustaceans significantly improves antioxidant capacity (AOC), immune function, stress resistance, and pigmentation, as evidenced in Pacific white shrimp (*Litopenaeus vannamei*), swimming crab (*Portunus trituberculatus*), and Chinese mitten crab (*Eriocheir sinensis*) [4–6]. However, the efficacy of synthetic carotenoids in improving antioxidant function remains inferior to that of natural pigments [7]. Microalgae, as ideal functional feed additives, combine the comprehensive advantages of natural origin, high efficacy, and low environmental impact [8, 9]. They not only grow rapidly with high yields but are also regarded as strategic resources for carotenoid production [10], exhibiting high bioactivity and absorption efficiency [11]. Furthermore, they provide proteins, lipids, polysaccharides, and other micronutrients, offering potential synergistic benefits beyond pigment supply. For instance, *Arthrospira platensis* is more effective than lutein in enhancing the AOC and disease resistance of aquatic animals [12]. Compared to synthetic astaxanthin, *Haematococcus pluvialis* powder is more efficient in enhancing the growth and AOC of *L. vannamei* [13].

*Dunaliella salina*, a halophilic green microalga, stands out as one of the richest natural sources of β‐carotene, comprising 8%–14% of its dry weight [14]. *D. salina* exhibits tolerance to extreme salinity, which not only reduces the risk of contamination during cultivation but also enables efficient utilization of seawater, salt lake water, and even industrial wastewater [15]. Its growth process can sequester carbon dioxide and produce high‐value natural β‐carotene [16]. Therefore, compared to the stringent cultivation conditions of *H. pluvialis*, *D. salina* offers a more cost‐effective and environmentally friendly alternative. Liu et al. [17] revealed through comparative transcriptomics that both astaxanthin from *H. pluvialis* and β‐carotene from *D. salina* improve the AOC and immunity of *E. sinensis*. Thus, *D. salina* can be considered a highly promising feed additive. Studies on *D. salina* in *L. vannamei* have confirmed its ability to significantly enhance immunity and AOC [18]. In addition, in black tiger shrimp (*Penaeus monodon*) and red claw crayfish (*Cherax quadricarinatus*), supplementation with *D. salina* powder has been shown to significantly improve body pigmentation [19, 20].

While the potential of *D. salina* is evident, critical knowledge gaps remain regarding its application in the farming of red swamp crayfish. First, the optimal dietary supplementation level required to effectively enhance antioxidant and immune functions in *P. clarkii* has not been systematically determined. Second, the quantitative relationship between dietary *D. salina*, carotenoid deposition, and body color parameters remains unclear. Most existing studies report qualitative improvements but lack the precise data needed for feed formulation. Third, it is still uncertain whether supplementation with *D. salina* can confer additional, previously unexplored benefits to *P. clarkii*, such as the promotion of ovarian development or enhanced stress tolerance as reported in prior studies [21, 22]. To address these gaps, this study systematically evaluated the effects of graded levels of *D. salina* powder on the growth performance, digestive enzyme activity, nonspecific immunity, AOC, air‐exposure stress resilience, tissue pigmentation, and carotenoid deposition in juvenile red swamp crayfish. The objectives were as follows: (1) to determine the optimal supplementation level for growth, health, and pigmentation; (2) to elucidate the correlations between carotenoid deposition and body color performance; and (3) to explore its potential novel benefits. The findings are expected to provide a scientific basis for the precise, value‐added use of *D. salina* in crayfish feeds, thereby supporting the sustainable and high‐quality development of the industry.

## 2. Materials and Methods

### 2.1. Feed Preparation Procedures

The feed formula was based on the balanced nutritional structure, and the proportion of *D. salina* powder was gradually increased to explore its suitable dosage in crayfish feed. Experimental diets were formulated by supplementing a basal formulation with *D. salina* powder at graded levels of 0.00%, 0.34%, 0.67%, 1.34%, and 2.00%, designated as D0, D0.34, D0.67, D1.34, and D2.00, respectively (Table 1). The basal diet composition and preparation process followed our previous study [3]. All solid feed ingredients were crushed using a high‐speed grinder and sieved through a 0.25 mm mesh to ensure homogeneity. The ingredients were then precisely weighed, thoroughly blended, and moistened with 20% distilled water to facilitate pelleting. Diets were extruded through a pelletizer to produce pellets measuring 2 mm in diameter and 10 mm in length. The formulated diets were subsequently air‐dried, vacuum‐sealed, and stored at −20°C in darkness to prevent nutrient degradation until use.

**Table 1:** Proximate and carotenoid composition, and *D. salina* powder supplementation amounts of the five experimental feeds used in the study.

Items	D0	D0.34	D0.67	D1.34	D2.00
Ingredients (%)					
* D. salina* powder	0.00	0.34	0.67	1.34	2.00
Wheat flour	14.2	13.86	13.53	12.86	12.20
Other ingredients^1^	85.8	85.8	85.8	85.8	85.8
Total	100	100	100	100	100
Composition (% dry diet)^2^					
Moisture	10.92	9.02	9.31	10.01	9.63
Crude protein	38.90	39.90	39.76	39.64	39.85
Crude lipid	7.29	7.37	7.72	7.20	7.37
Ash	11.26	11.19	11.60	11.61	12.29
Carotenoid content (mg kg ^−1^ dry diet)^3^					
Total carotenoids	4.21 ± 1.09^d^	65.98 ± 2.45^c^	162.96 ± 0.73^b^	259.41 ± 16.71^b^	402.78 ± 22.44^a^
Astaxanthin	0.97 ± 0.07^c^	4.96 ± 0.07^b^	5.37 ± 0.54 b^b^	10.68 ± 0.50^a^	15.36 ± 1.10^a^
Lutein	1.54 ± 0.06^e^	2.79 ± 0.04^d^	5.55 ± 0.07^c^	6.35 ± 0.16^b^	10.89 ± 0.12^a^
Zeaxanthin	0.35 ± 0.03^d^	5.48 ± 0.10^c^	8.78 ± 0.22^b^	23.77 ± 5.27^a,b,c,d^	33.34 ± 0.26^a^
β‐Carotene	1.32 ± 0.29^d^	48.01 ± 0.40^c^	104.36 ± 1.04^b^	206.90 ± 16.76^a,b^	299.00 ± 7.59^a^

*Note:* Within a row, distinct superscript letters denote significant differences (*p* < 0.05).

^1^Other ingredients included fish meal, soybean meal, rapeseed meal, peanut meal, fish soluble, fish oil, vitamin premix, mineral premix, and so on, as detailed in Zhang et al. [3].

^2^The determination and analysis were carried out in accordance with the standard procedures [23, 24].

^3^Carotenoid data are mean ± SE (*n* = 3).

### 2.2. Experimental Design

This study comprised a 60‐day feeding trial with different *D. salina* powder‐supplemented diets and a 24 h air‐exposure stress test. Juvenile red swamp crayfish were collected from the Chongming Aquaculture Demonstration Base affiliated with Shanghai Ocean University. Before the formal experiment, all crayfish were subjected to a 7‐day acclimation period. Two hundred forty juvenile crayfish (initial average weight 6.28 g, sex ratio = 1 : 1) that were similar in size, vigorous, and morphologically intact were selected and randomly stocked into five dietary groups, each with three recirculating aquaculture tank replicates. The tanks were 224 mm in length, 140 mm in width, and 330 mm in height, with a water level maintained at 200 mm, and were partitioned using plastic dividers. Sixteen juvenile crayfish were evenly distributed in each tank. Daily rations (3%–5% of the total body weight) were dispensed at 18 : 00, residues and feces were siphoned off at 08 : 00 to safeguard water quality. If any crayfish died during the experiment, the dead individuals were immediately removed to prevent water contamination. Crayfish were exposed to a 12L:12D cycle for the entire study, and water quality parameters were kept within the optimal range. At the termination of the feeding trial, four crayfish per replicate were retained for the subsequent air‐exposure challenge. The body weight and length of the crayfish were measured using a digital balance and a vernier caliper. The weights of the tail muscles, hepatopancreas, and ovaries were also recorded. Subsequently, the following indices were calculated as follows:

Survival rate SR;%=N/48100×;

Condition factor CF=W/L3×100;

Weight gain rate WGR;%=W–Wo/Wo×100;

Specific growth rate SGR;%/d=lnW– lnWo/60100×;

Hepatosomatic index HSI;%=H/W×100;

Gonadosomatic index GSI;%=O/W×100;

Meat yield MY;%=M/W×100.

where *N* represents the number of surviving crayfish in each group at the end of the experiment, *Wo*, *W*, and *L* denote the initial body weight, final body weight, and body length, respectively, while *H*, *M*, and *O* are the hepatopancreas, muscle, and ovary tissue weights, respectively.

The remaining crayfish were individually transferred to clean plastic containers (115 mm × 85 mm × 60 mm) and subjected to a 24‐h air exposure stress test. Throughout the test period, environmental conditions were strictly controlled, with temperature maintained at 20 ± 1°C and relative humidity at 50 ± 5%.

### 2.3. Sample Collection and Determination of Enzyme Activities

After trials were completed, the crayfish were anesthetized with ice water, measured and weighed, then dissected for sampling. The hemolymph and hepatopancreas samples from the feeding trial, as well as the hepatopancreas and muscle tissues from the stress test, were immediately stored in nine volumes of ice‐cold physiological saline solution, homogenized, centrifuged, and the supernatant was obtained for subsequent analysis. All enzyme activity determinations were strictly carried out in accordance with the kits provided by the manufacturer (Jiancheng Bioengineering Institute, Nanjing, China). Four digestive enzymes, including lipase (LPS), α‐amylase (α‐AMS), pepsin (PEP), and cellulase (CL), as well as immune and antioxidant indices such as acid phosphatase (ACP), alkaline phosphatase (AKP), total superoxide dismutase (T‐SOD), total AOC (T‐AOC), and malondialdehyde (MDA), were determined. In addition, respiratory metabolism‐related indicators, including MDA, lactate (LD), and succinate dehydrogenase (SDH) after air exposure stress, were evaluated [25].

### 2.4. Color Parameters Determination

The surface color of freeze–dried carapace, muscle, hepatopancreas, and ovary samples was measured using a CR‐400 colorimeter (Konica Minolta, Marunouchi, Tokyo, Japan), which provided *L* ^∗^ (lightness), *a* ^∗^ (red–green), and *b* ^∗^ (yellow–blue) values [26]. Six random measurements were taken per sample and averaged for data analysis.

### 2.5. Carotenoid Composition Assessment

Total carotenoid content in diets and tissues was extracted with acetone and quantified spectrophotometrically at 470 nm. Individual carotenoids (astaxanthin, lutein, zeaxanthin, canthaxanthin, and β‐carotene) were identified and quantified using high‐performance liquid chromatography (HPLC) following established methods [3, 27]. The actual β‐carotene content in feeds was 1.32, 48.01, 104.36, 206.90, and 299.00 mg kg^−1^, respectively. The dietary carotenoid content was linearly correlated with the supplementation level of *D. salina* powder (Figure 1).

Figure 1Dose–response linearity between *D. salina* powder inclusion and dietary carotenoid concentration. (a) Total carotenoids; (b) astaxanthin; (c) lutein; (d) zeaxanthin; (e) β‐carotene. Each point is the mean ± SE of three independent tanks.

**Figure:**
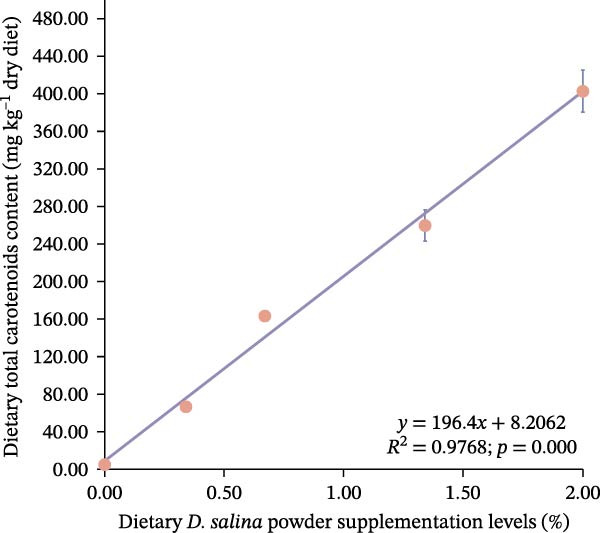
(a)

**Figure:**
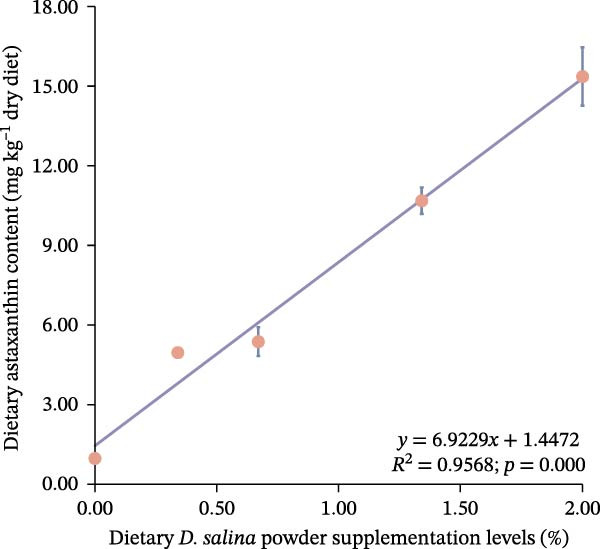
(b)

**Figure:**
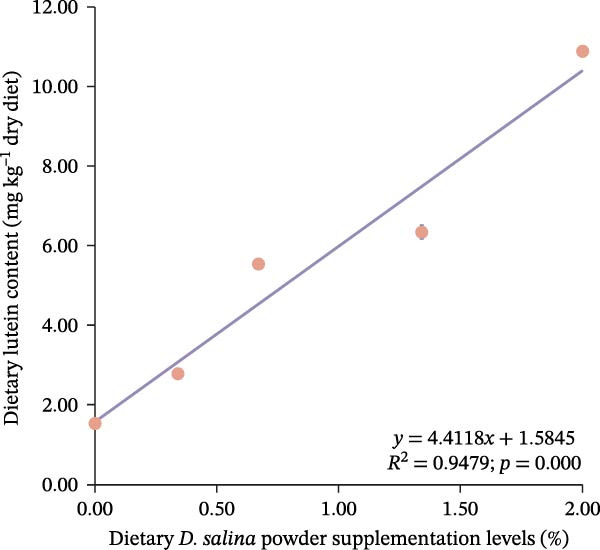
(c)

**Figure:**
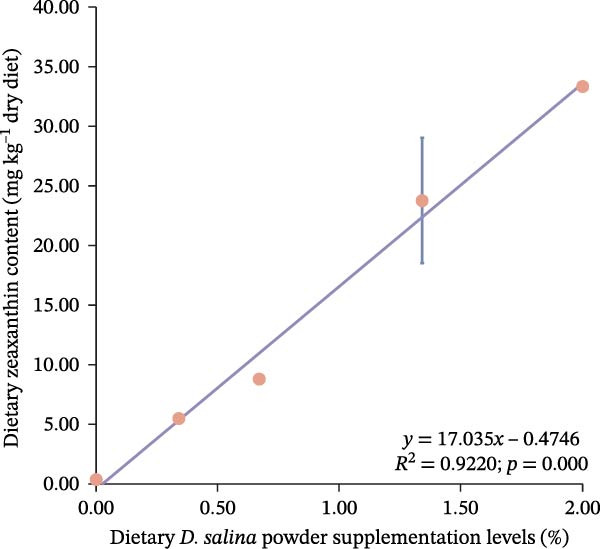
(d)

**Figure:**
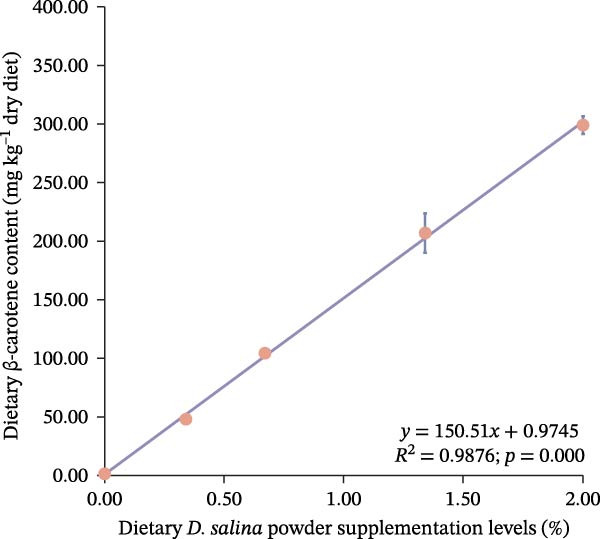
(e)

### 2.6. Statistical Analysis

The Shapiro–Wilk test was applied to confirm normal distribution, and equality of variances was checked with Levene’s test. For data meeting both assumptions, differences among groups were assessed by one‐way ANOVA followed by Duncan’s post hoc comparisons; otherwise, Welch’s test and the Games–Howell comparison test were applied. Regression analysis was conducted to evaluate potential linear or quadratic relationships between the dietary *D. salina* powder supplementation levels and the corresponding response variables. Relationships among carotenoid content in the diets and crayfish tissues, color parameters, and physiological indicators were explored using Pearson correlation analysis. The optimal dietary supplementation level of *D. salina* powder was determined based on carotenoid deposition in crayfish tissues. Analyses were performed with IBM SPSS Statistics v22.0, bar plots were generated in GraphPad Prism 9, and correlation heatmaps were constructed with Origin 2023.

## 3. Results

### 3.1. Survival and Growth Indices

After the *D. salina* powder supplemented diets feeding trial, except for the D0 group (95.24%) and D0.34 group (97.62%), survival in all other treatment groups reached 100%. Growth performance parameters of crayfish fed diets containing different amounts of *D. salina* powder are presented in Table 2. Both weight gain rate (WGR) and specific growth rate (SGR) exhibited a nonlinear response to dietary *D. salina* inclusion, declining to their lowest values in the D0.34 group before increasing to a maximum in the D2.00 group. Gonadosomatic index (GSI) in D0.34 exceeded that in all other groups (*p* < 0.05). Furthermore, no differences were detected in FBW, CF, MY, and HSI among the treatment groups (*p* > 0.05).

**Table 2:** Growth performance parameters of juvenile crayfish fed diets supplemented different *D. salina* powder levels.

Items	D0	D0.34	D0.67	D1.34	D2.00	Linear		Quadratic	
						*p*	*R* ^2^	*p*	*R* ^2^
IBW (g)	5.99 ± 0.25	6.62 ± 0.20	6.48 ± 0.09	6.23 ± 0.32	6.10 ± 0.22	0.637	0.018	0.333	0.167
FBW (g)	12.30 ± 0.74	10.83 ± 0.68	12.42 ± 1.43	13.54 ± 1.07	14.03 ± 0.98	0.066	0.524	0.372	0.490
CF (%)	6.58 ± 0.17	6.54 ± 0.07	6.57 ± 0.21	6.56 ± 0.05	6.38 ± 0.13	0.310	0.079	0.499	0.109
WGR (%)	106.69 ± 15.35^a,b^	71.19 ± 13.41^b^	108.49 ± 15.84^a,b^	124.48 ± 17.89^a^	134.43 ± 9.06^a^	0.027	0.178	0.215	0.178
SGR (%/d)	1.19 ± 0.13^a,b^	0.87 ± 0.12^b^	1.20 ± 0.13^a,b^	1.32 ± 0.14^a^	1.41 ± 0.06^a^	0.027	0.148	0.191	0.155
HSI (%)	6.90 ± 0.34	7.00 ± 0.18	7.07 ± 0.16	6.94 ± 0.47	7.57 ± 0.21	0.145	0.156	0.264	0.199
GSI (%)	2.57 ± 0.19^b,c^	4.04 ± 0.56^a^	3.39 ± 0.30^a,b^	2.13 ± 0.28^c^	2.35 ± 0.23^b,c^	0.093	0.201	0.181	0.248
MY (%)	13.72 ± 0.35	14.14 ± 0.13	12.87 ± 0.38	13.59 ± 0.44	13.18 ± 0.53	0.300	0.082	0.560	0.092

*Note*: Data of carotenoid content are expressed mean ± SE (*n* = 3), in the same row, data with distinct superscript letters indicate differences significant at *p*  < 0.05.

### 3.2. Digestive Enzymes

Figure 2 depicts the digestive enzyme profile of juvenile crayfish. All four digestive enzymes exhibited a single‐peak pattern, with activities highest in the D0.34 group. Specifically, α‐AMS, PEP, and CL activities in D0.34 exceeded those of D0, D1.34, and D2.00 (*p* < 0.05), whereas LPS displayed a significant quadratic relationship with dietary *D. salina* level (*p* < 0.05).

Figure 2Digestive response parameters to dietary *D. salina* powder levels of crayfish. Results are presented as mean ± SE (*n* = 3), within five treatments, bars topped by different letters differ significantly (*p* < 0.05): (a) Lipase; (b) α‐amylase; (c) Pepsin; (d) Cellulase.

**Figure:**
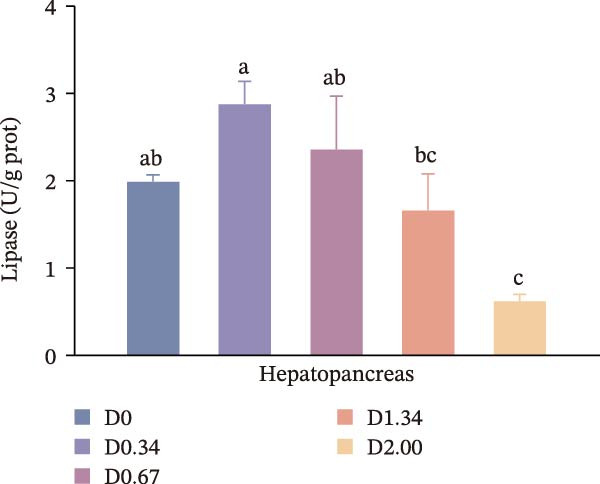
(a)

**Figure:**
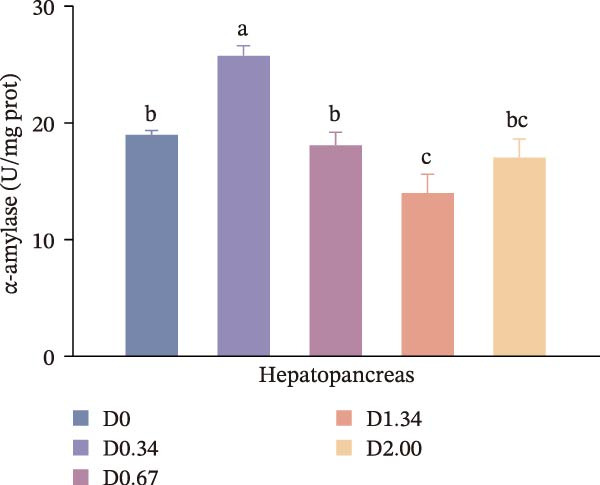
(b)

**Figure:**
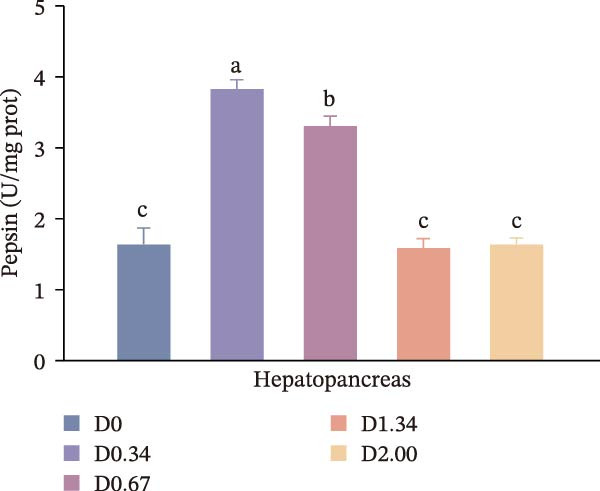
(c)

**Figure:**
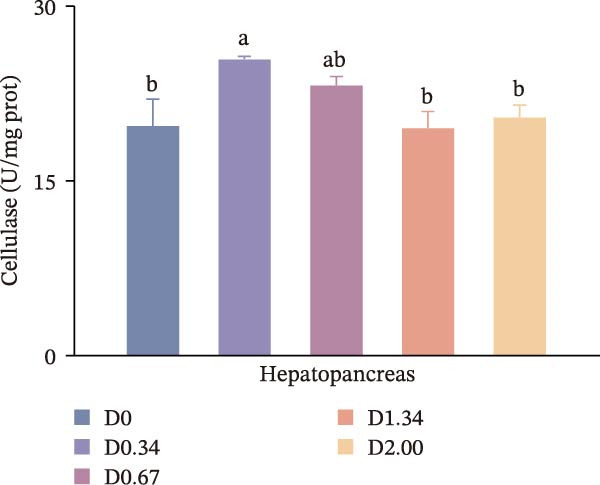
(d)

### 3.3. Nonspecific Immune Enzymes

As shown in Figure 3, in the hepatopancreas, AKP activity demonstrated a significant quadratic relationship with the level of *D. salina* powder (*p* < 0.05), peaking in the D0.67 group, surpassing all other treatments (*p* < 0.05). However, ACP activity remained comparable among groups (*p* > 0.05). In the hemolymph, AKP activity was the highest in the D0.34 group (*p* < 0.05). With rising *D. salina* inclusion, ACP activity gradually rose, peaking in the D1.34 group, then declined, and exhibited a significant quadratic correlation with the *D. salina* powder levels.

Figure 3Nonspecific immune response parameters to dietary *D. salina* powder levels of crayfish. (a, c) AKP, alkaline phosphatase; (b, d) ACP, acid phosphatase. Results are presented as mean ± SE (*n* = 3), within five treatments, bars topped by different letters differ significantly (*p* < 0.05): (a) AKP, alkaline phosphatase in the hepatopancreas; (b) ACP, acid phosphatase in the hepatopancreas; (c) AKP, alkaline phosphatase in the hemolymph; (d) ACP, acid phosphatase in the hemolymph.

**Figure:**
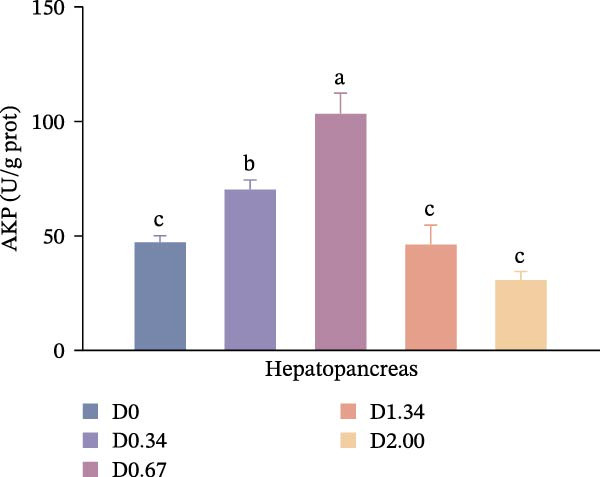
(a)

**Figure:**
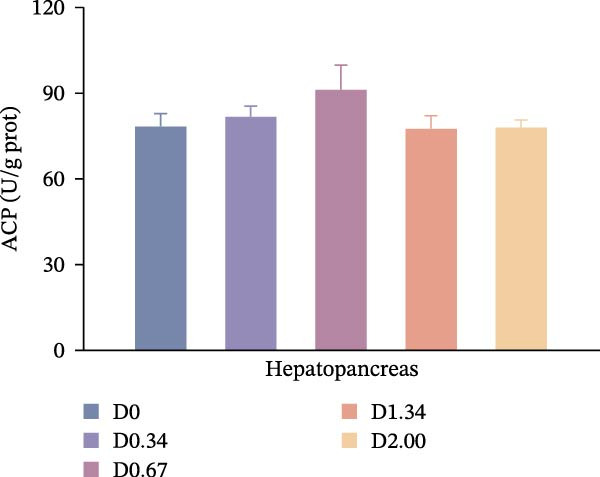
(b)

**Figure:**
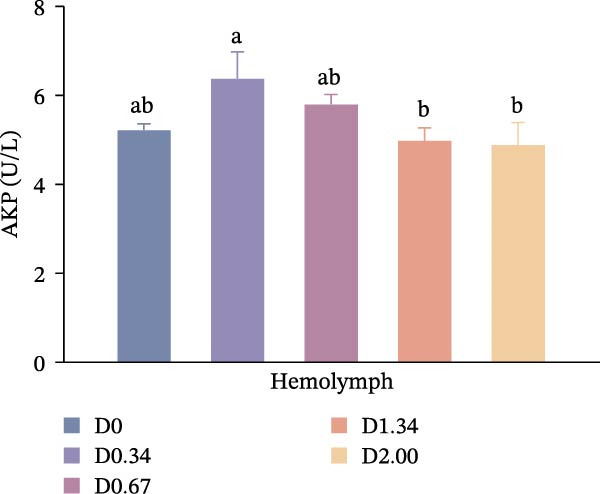
(c)

**Figure:**
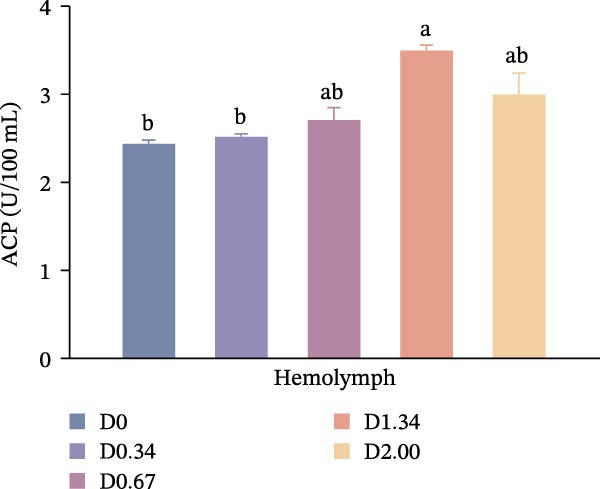
(d)

### 3.4. Antioxidative Response

The antioxidative response‐related parameters are presented in Figure 4. T‐SOD activity in the hepatopancreas and hemolymph, as well as T‐AOC in the hemolymph, first increased and then declined with rising *D. salina* powder amounts, reaching its peak in the D0.67 group, remarkably exceeding the D0 level (*p* < 0.05). However, no significant differences emerged in T‐AOC in the hemolymph between the control group and other *D. salina* supplemented groups (*p* > 0.05). Hepatopancreatic and hemolymph MDA levels of *D. salina* powder‐supplemented groups fell significantly below D0 values (*p* < 0.05), reaching their lowest values in the D2.00 and D0.34 groups, respectively. Moreover, regression analysis indicated that hepatopancreatic MDA and T‐AOC, along with hemolymph T‐SOD activity, showed significant quadratic correlations with the level of *D. salina* powder (*p* < 0.05).

Figure 4Antioxidant response parameters to dietary *D. salina* powder levels of crayfish. (a) T‐SOD, total superoxide dismutase; (b) T‐AOC, total antioxidant capacity; (c) MDA, malondialdehyde. Results are shown as mean ± SE (*n* = 3), bars topped by different letters differ among the five treatments (*p* < 0.05).

**Figure:**
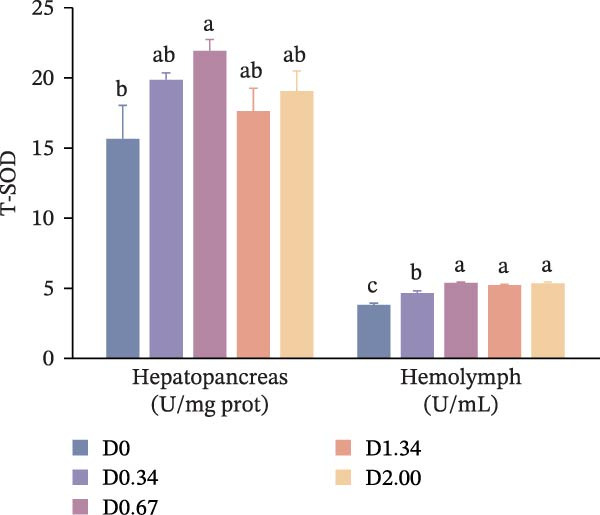
(a)

**Figure:**
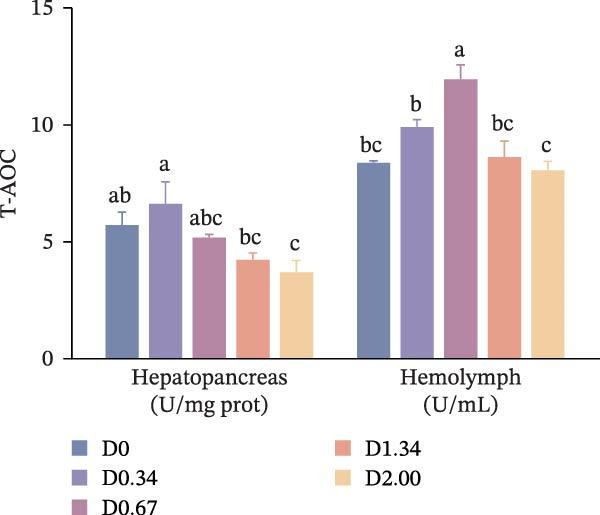
(b)

**Figure:**
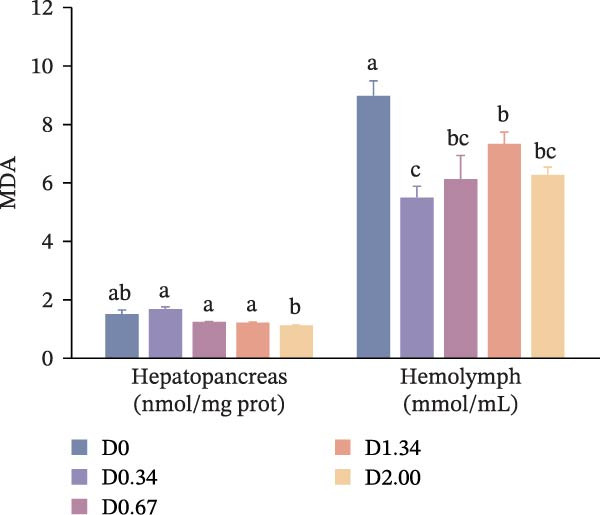
(c)

### 3.5. Air Exposure Stress Tolerance

After 24 h exposure to air stress, stress tolerance‐related parameters are shown in Figure 5. Hepatopancreatic and muscle MDA levels of crayfish decreased with the increase of the level of *D. salina* powder, reaching its lowest value in the D2.00 group, which exhibited a quadratic relationship with dietary *D. salina* amounts (*p* < 0.05). Muscle LD content dropped steadily, and the lowest value also appeared in the D2.00 group, falling below D0 group (*p* < 0.05), whereas hepatopancreatic LD remained uniform across groups (*p* > 0.05). The activity of SDH in the hepatopancreas initially increased and then decreased, reaching the peak in the D0.34 group (*p* < 0.05). In contrast, SDH activity in the muscles rose continuously to a maximum in D2.00 group, considerably correlated with the level of *D. salina* supplementation (*p* < 0.05).

Figure 5Air‐exposure stress response parameters to dietary *D. salina* powder levels of crayfish. (a) MDA, malondialdehyde; (b) LD, lactic acid; (c) SDH, succinate dehydrogenase. Results are shown as mean ± SE (*n* = 3), bars topped by different letters differ among the five treatments (*p* < 0.05).

**Figure:**
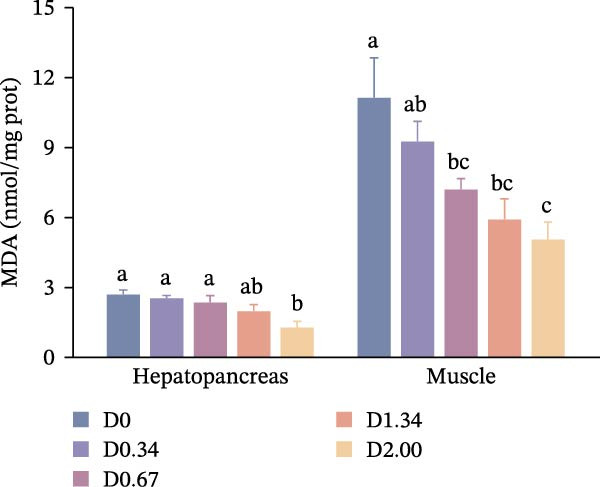
(a)

**Figure:**
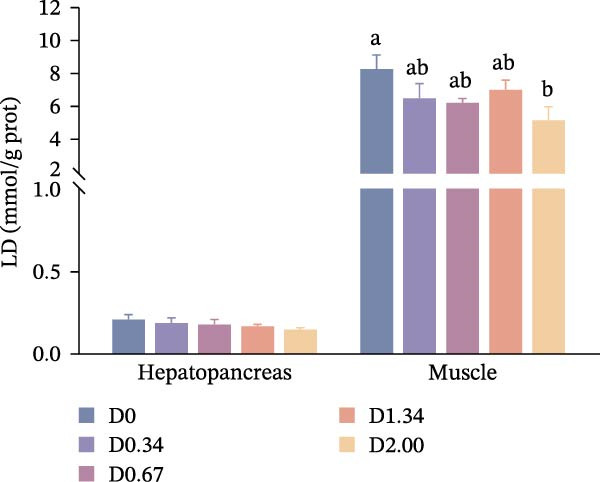
(b)

**Figure:**
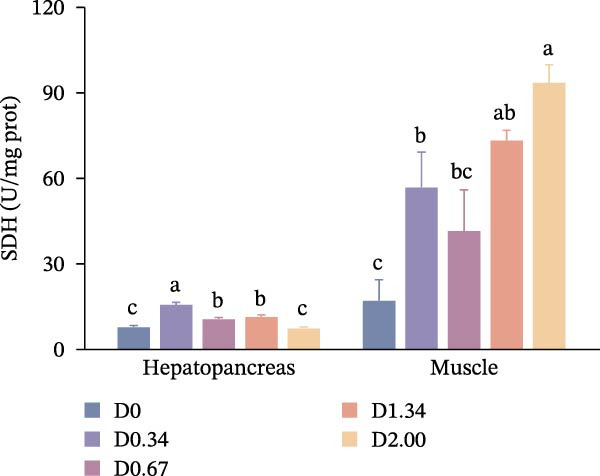
(c)

### 3.6. Coloration Parameters

Table 3 presents the color values of different tissues (carapaces, muscles, hepatopancreas, and ovaries). The *L* ^∗^ and *a* ^∗^ values in the carapaces of crayfish fed with *D. salina* powder supplemented diets were pronouncedly lower/higher than those of the D0 group (*p* < 0.05), with extreme values observed in the D1.34 group, with a significant quadratic relationship with the levels of *D. salina* powder (*p* < 0.05). The *b* ^∗^ value of the carapaces exhibited a quadratic response to dietary *D. salina* powder (*p* < 0.05), initially declining and then rising, with the minimum recorded in the D0.67 group. The *L* ^∗^ and *a* ^∗^ values in the muscles were consistent with the trend in the carapaces, while the *b* ^∗^ value showed the opposite trend, and both showed significant quadratic changes (*p* < 0.05). Similarly, hepatopancreatic and ovarian *L* ^∗^ values continuously decreased, with the lowest values occurring in the D0.34 and D0.67 groups, respectively, and the *b* ^∗^ value in the ovaries showed the opposite trend, peaking in the D1.34 group, and all differed markedly from the control (*p* < 0.05). No significant effects of *D. salina* powder were detected on hepatopancreatic *a* ^∗^ or *b* ^∗^, or on ovarian *a* ^∗^, relative to the D0 group (*p* > 0.05). β‐Carotene from *D. salina* produced tissue‐dependent color shifts in crayfish.

**Table 3:** Effects of dietary *D. salina* powder levels on the pigmentation traits of juvenile crayfish.

Items		D0	D0.34	D0.67	D1.34	D2.00	Linear		Quadratic	
							*p*	*R* ^2^	*p*	*R* ^2^
*L* ^∗^	Carapaces	54.84 ± 0.34^a^	48.23 ± 0.86^b^	46.16 ± 0.50^b^	45.30 ± 0.76^b^	47.17 ± 0.51^b^	0.012	0.395	0.000	0.880
*a* ^∗^	Carapaces	20.82 ± 1.29^b^	28.73 ± 0.89^a^	30.51 ± 0.73^a^	31.10 ± 0.37^a^	30.14 ± 0.59^a^	0.009	0.418	0.000	0.814
*b* ^∗^	Carapaces	24.53 ± 0.57^a^	24.23 ± 0.40^a,b^	22.99 ± 0.31^b,c^	22.30 ± 0.42^c^	23.46 ± 0.18^a,b,c^	0.055	0.255	0.004	0.595
*L* ^∗^	Muscle	91.92 ± 0.32^a^	90.23 ± 0.11^c^	90.43 ± 0.27^b,c^	90.10 ± 0.35^c^	91.36 ± 0.49^a,b^	0.727	0.010	0.005	0.592
*a* ^∗^	Muscle	2.70 ± 0.17^b^	4.06 ± 0.19^a^	3.49 ± 0.33^a,b^	3.18 ± 0.13^a,b^	2.63 ± 0.16^b^	0.218	0.114	0.036	0.425
*b* ^∗^	Muscle	5.97 ± 0.21^b^	6.79 ± 0.02^a,b^	8.47 ± 0.34^a^	8.40 ± 0.70^a,b^	6.99 ± 0.32^a,b^	0.215	0.116	0.001	0.703
*L* ^∗^	Hepatopancreas	34.66 ± 0.39^a^	30.07 ± 1.54^b^	34.53 ± 1.85^a^	32.92 ± 0.79^a,b^	32.69 ± 0.64^a,b^	0.809	0.005	0.946	0.009
*a* ^∗^	Hepatopancreas	8.42 ± 0.89	7.93 ± 1.17	10.34 ± 0.60	8.96 ± 0.78	7.37 ± 0.81	0.510	0.034	0.187	0.244
*b* ^∗^	Hepatopancreas	12.42 ± 0.44^a,b,c^	11.52 ± 0.54^b,c^	14.78 ± 1.06^a^	10.09 ± 1.37^c^	13.27 ± 0.80^a,b^	0.952	0.000	0.926	0.013
*L* ^∗^	Ovaries	55.05 ± 0.34^a^	52.83± 0.49^a,b^	48.81 ± 0.54^c^	53.44 ± 0.41^a^	49.74 ± 0.57^b,c^	0.060	0.246	0.117	0.301
*a* ^∗^	Ovaries	24.88 ± 0.14	25.12 ± 0.20	25.30 ± 0.68	25.17 ± 0.27	25.13 ± 0.32	0.704	0.011	0.744	0.048
*b* ^∗^	Ovaries	19.10 ± 0.32^b^	20.52 ± 0.12^a,b^	18.95 ± 0.61^a,b^	22.18 ± 0.27^a^	19.71 ± 0.33^b^	0.267	0.094	0.143	0.277

*Note:* Values are presented as mean ± SE (*n* = 3), within each row, distinct superscript letters denote differences significant at *p*  < 0.05.

### 3.7. Carotenoid Composition

The accumulation contents of carotenoids are presented in Table 4. In the carapaces, the contents of total carotenoids, astaxanthin, and lutein showed a general increasing trend with dietary *D. salina* level, attaining their highest concentrations in the D1.34 and D2.00 groups. Zeaxanthin and β‐carotene peaked in the D0.34 and D0.67 groups, respectively, and were significantly higher than in the control group (*p* < 0.05). In the muscles, zeaxanthin content was the highest in the D0.34 group, and β‐carotene content was the highest in the D0.67 group, both significantly higher than the control group (*p* < 0.05). In the ovaries, zeaxanthin and β‐carotene continuously increased with the supplementation level of *D. salina* powder, reaching their maximum values in the D1.34 group (*p* < 0.05). Similarly, astaxanthin and zeaxanthin contents also increased and reached their peaks in the D0.67 and D2.00 groups, respectively, and both were significantly higher than the control group (*p* < 0.05). However, although total carotenoids in the D1.34 group and lutein in the D2.00 group were numerically highest, neither differed significantly from the control (*p* > 0.05). Additionally, the contents of other measured carotenoids did not show significant differences among the groups (*p* > 0.05). Notably, astaxanthin content in the carapaces, as well as lutein and zeaxanthin contents in the ovaries, exhibited a significant linear increase in relation to the dietary *D. salina* powder content (*p* < 0.05) (Figure 6). Based on quadratic regression models, it was determined that supplementing the diet with 1.23%–1.53% *D. salina* powder resulted in the highest levels of total carotenoids and lutein in the carapaces, as well as total carotenoids and β‐carotene in the ovaries (Figure 7).

Figure 6Linear relationship of carotenoid contents in tissues to *D. salina* powder supplementation levels. (a) Astaxanthin in carapaces; (b) lutein in ovaries; (c) zeaxanthin in ovaries. Each point is the mean ± SE of three independent tanks.

**Figure:**
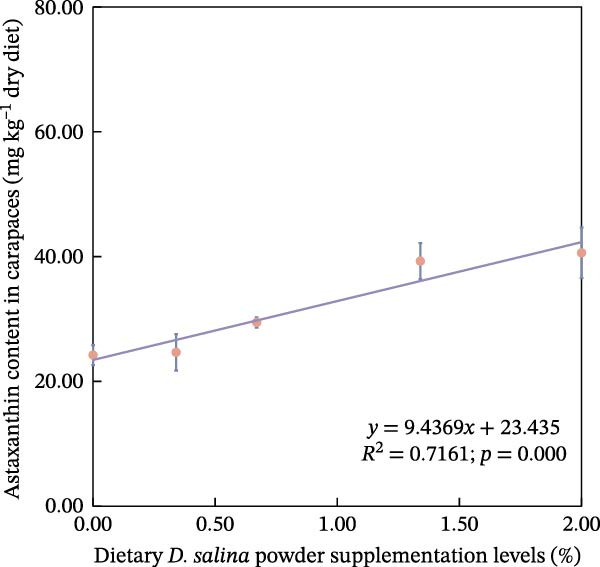
(a)

**Figure:**
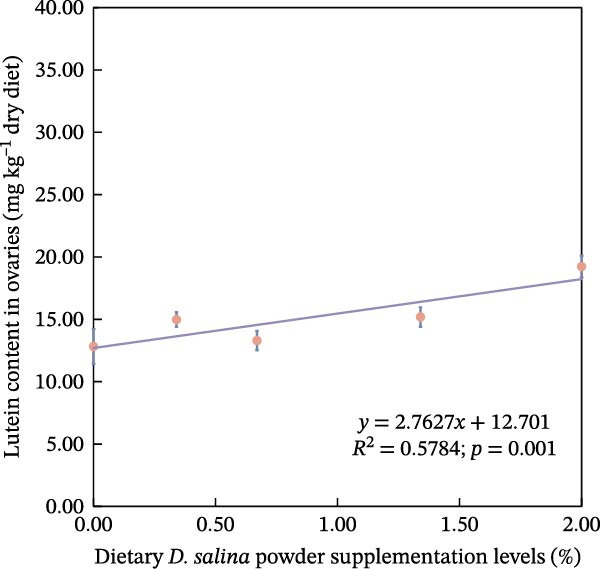
(b)

**Figure:**
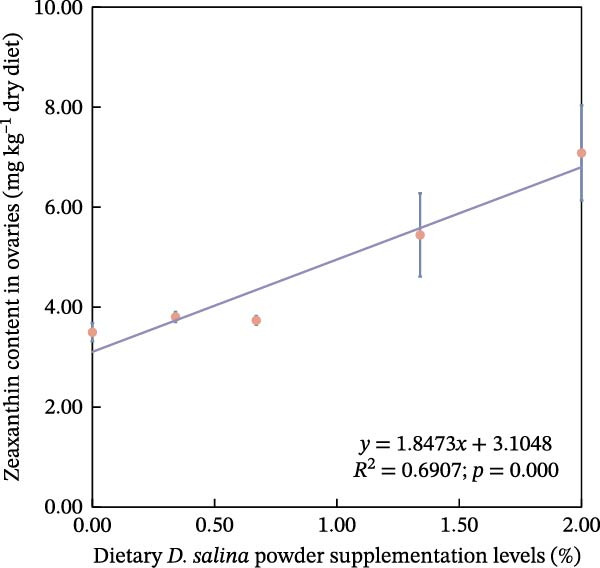
(c)

Figure 7Regression analysis of dietary *D. salina* powder supplementation levels based on carotenoid accumulation in tissues of red swamp crayfish. (a) Total carotenoids in carapaces; (b) lutein in carapaces; (c) total carotenoids in ovaries; (d) β‐carotene in ovaries. Each point is the mean ± SE of three independent tanks.

**Figure:**
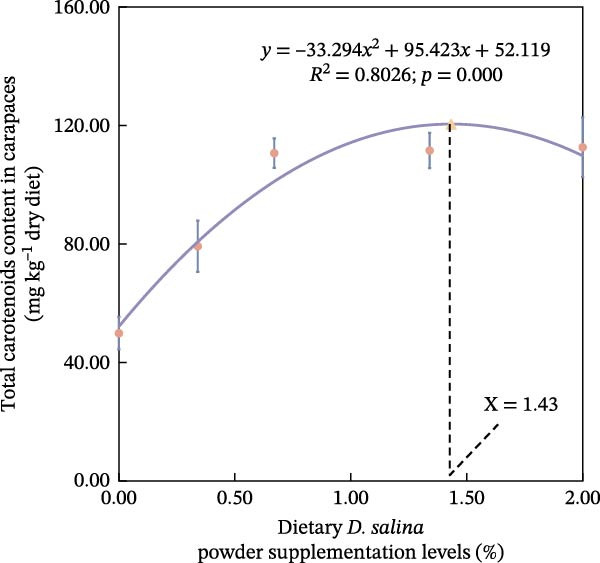
(a)

**Figure:**
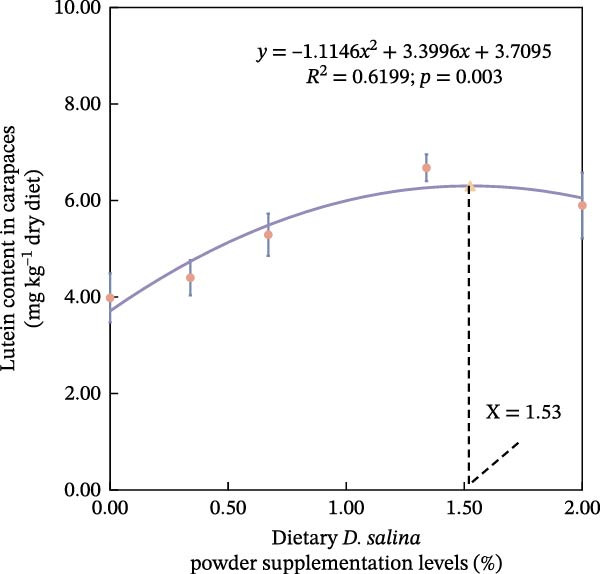
(b)

**Figure:**
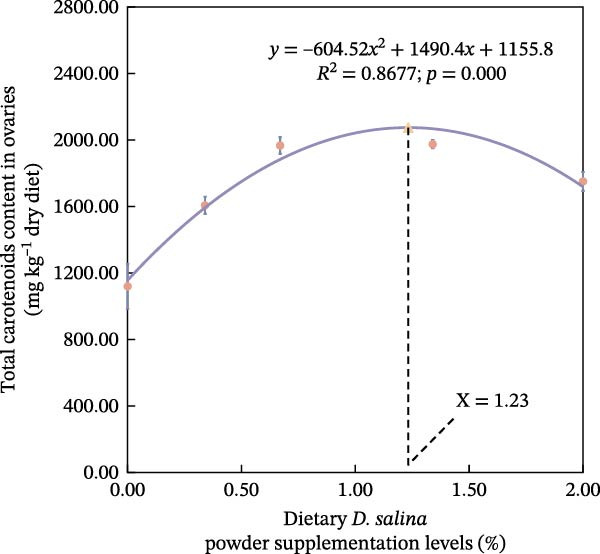
(c)

**Figure:**
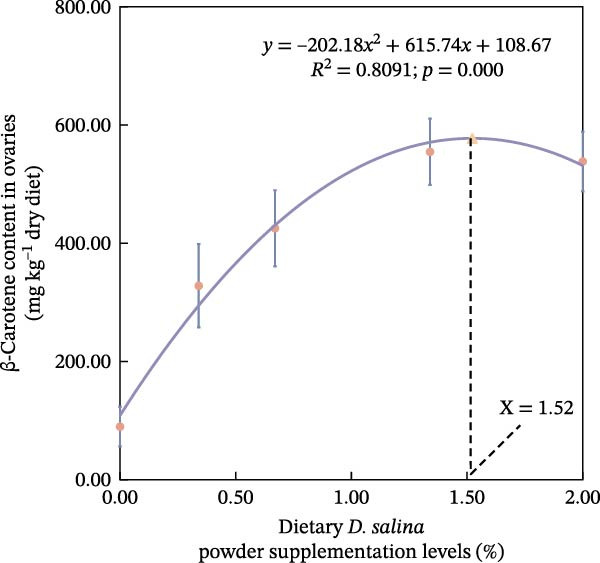
(d)

**Table 4:** Effects of dietary *D. salina* powder levels on carotenoid deposition of juvenile crayfish (dry matter) mg kg^−1^.

Items		D0	D0.34	D0.67	D1.34	D2.00	Linear		Quadratic	
							*p*	*R* ^2^	*p*	*R* ^2^
Total carotenoids	Carapaces	49.87 ± 5.48^b^	79.22 ± 8.63^a,b^	110.65 ± 4.99^a^	111.54 ± 5.97^a^	112.65 ± 10.09^a^	0.001	0.559	0.000	0.803
Astaxanthin	Carapaces	24.22 ± 1.61^b^	24.67 ± 2.92^b^	29.45 ± 0.82^b^	39.28 ± 2.89^a^	40.60 ± 4.06^a^	0.000	0.716	0.000	0.724
Lutein	Carapaces	3.98 ± 0.51^c^	4.40 ± 0.37^b,c^	5.29 ± 0.44^a,b,c^	6.68 ± 0.28^a^	5.90 ± 0.68^a,b^	0.004	0.479	0.003	0.620
Zeaxanthin	Carapaces	0.50 ± 0.09^a,b^	0.53 ± 0.08^a,b^	0.53 ± 0.04^b^	0.76 ± 0.02^a^	0.62 ± 0.08^a,b^	0.054	0.257	0.088	0.333
Canthaxanthin	Carapaces	9.07 ± 0.04^b^	9.21 ± 0.04^a^	9.17 ± 0.02^a,b^	9.14 ± 0.02^a,b^	9.17 ± 0.04^a,b^	0.446	0.045	0.451	0.124
β‐carotene	Carapaces	11.67 ± 0.21^c^	12.28 ± 0.22^b,c^	13.49 ± 0.53^a^	12.85 ± 0.21^a,b^	13.05 ± 0.38^a,b^	0.057	0.251	0.027	0.452
Total carotenoids	Muscle	14.85 ± 1.13	19.67 ± 2.30	26.08 ± 7.47	19.45 ± 1.16	17.54 ± 3.92	0.953	0.000	0.280	0.191
Astaxanthin	Muscle	9.79 ± 0.20	10.40 ± 0.06	10.78 ± 1.12	9.66 ± 0.13	9.66 ± 0.77	0.460	0.043	0.535	0.099
Zeaxanthin	Muscle	0.40 ± 0.03^b,c^	0.71 ± 0.02^a^	0.56 ± 0.01^b^	0.54 ± 0.02^b^	0.49 ± 0.06^a,b^	0.769	0.007	0.173	0.254
β‐carotene	Muscle	9.89 ± 0.07^c^	10.29 ± 0.11^b,c^	10.96 ± 0.10^a^	10.92 ± 0.11^a,b^	10.66 ± 0.06^a,b^	0.017	0.366	0.000	0.841
Total carotenoids	Hepatopancreas	27.62 ± 5.42	24.57 ± 1.99	153.31 ± 41.74	67.04 ± 34.95	31.60 ± 6.39	0.993	0.000	0.101	0.317
Astaxanthin	Hepatopancreas	9.55 ± 0.12	9.70 ± 0.07	9.78 ± 0.18	9.85 ± 0.16	9.95 ± 0.14	0.037	0.294	0.106	0.312
β‐carotene	Hepatopancreas	15.69 ± 1.15	20.82 ± 0.65	100.60 ± 35.66	58.52 ± 28.89	23.51 ± 2.56	0.833	0.004	0.077	0.348
Total carotenoids	Ovaries	1119.36 ± 134.77^a,b^	1606.68 ± 51.34^b^	1966.45 ± 50.40^a^	1974.44 ± 23.95^a^	1750.54 ± 56.93^a,b^	0.024	0.335	0.000	0.868
Astaxanthin	Ovaries	507.64 ± 35.70^b^	659.84 ± 24.55^a^	737.02 ± 32.68^a^	650.96 ± 23.32^a^	678.14 ± 38.30^a^	0.116	0.179	0.022	0.471
Lutein	Ovaries	12.82 ± 1.39^a,b^	14.98 ± 0.59^a,b^	13.30 ± 0.78^b^	15.19 ± 0.78^a,b^	19.23 ± 0.87^a^	0.001	0.578	0.002	0.654
Zeaxanthin	Ovaries	3.50 ± 0.18^c^	3.80 ± 0.10^b,c^	3.73 ± 0.09^b,c^	5.44 ± 0.83^a,b^	7.08 ± 0.95^a^	0.000	0.691	0.000	0.729
Canthaxanthin	Ovaries	19.89 ± 0.06^b^	20.08 ± 0.02^a,b^	20.05 ± 0.08^a,b^	20.12 ± 0.06^a^	20.04 ± 0.06^a,b^	0.209	0.118	0.048	0.396
β‐carotene	Ovaries	89.73 ± 33.45^b^	328.02 ± 70.43^a,b^	425.25 ± 64.36^a,b^	554.68 ± 56.07^a^	538.27 ± 50.45^a^	0.000	0.624	0.000	0.809

*Note:* Values are presented as mean ± SE (*n* = 3), within each row, distinct superscript letters denote differences significant at *p*  < 0.05.

### 3.8. Correlation Analysis of Carotenoid Content With Tissue Color Parameters

**Figure 8:**
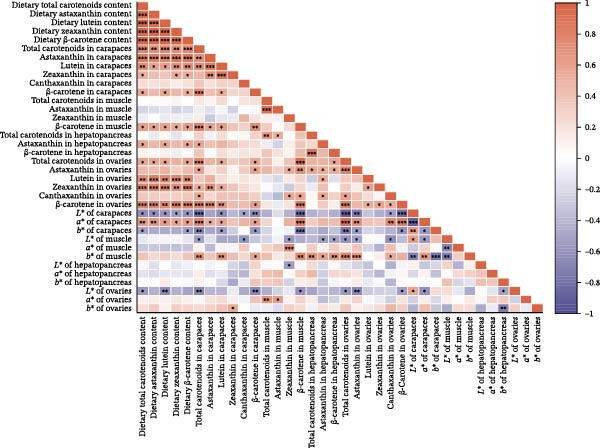
The heatmap of the relationships among dietary carotenoid content, color parameters, and carotenoid contents in different tissues of red swamp crayfish using the Pearson correlation analysis. Red bars are positive, purple bars are negative, and  ^∗^,  ^∗∗^, and  ^∗∗∗^ mark significance at *p*  < 0.05, *p*  < 0.01, and *p*  < 0.001, respectively.

The correlations between carotenoid contents and color parameters are illustrated in Figure 8. A highly significant correlation was observed among various carotenoids in the feed (*p* < 0.001). Carapace total carotenoids and astaxanthin, together with ovarian lutein, zeaxanthin, and β‐carotene content, all positively with dietary carotenoid level (*p* < 0.01). Furthermore, a significant positive correlation was observed among the various carotenoids within the carapaces and ovaries, respectively. More importantly, carotenoid accumulation was significantly correlated with shifts in tissue color parameters. To be specific, the *L* ^∗^, *b* ^∗^,and *a* ^∗^ values in the carapaces are significantly positively/negatively correlated with the total carotenoid content in the carapaces (*p* < 0.01). In the ovaries, the *L* ^∗^ value was significantly negatively correlated with total carotenoids, astaxanthin, and β‐carotene contents (*p* < 0.05). Additionally, the *L* ^∗^ value fell sharply with a rising *a* ^∗^ value in the carapaces (*p* < 0.001), while a significant positive correlation was observed between the *L* ^∗^ and *b* ^∗^ values (*p* < 0.01). In the muscle tissue, the *L* ^∗^ value was significantly negatively correlated with both the *a* ^∗^ and *b* ^∗^ values (*p* < 0.01), whereas the correlations in the hepatopancreas and ovaries were less pronounced.

### 3.9. Correlation Analysis of Carotenoid Content With Physiological Indicators

**Figure 9:**
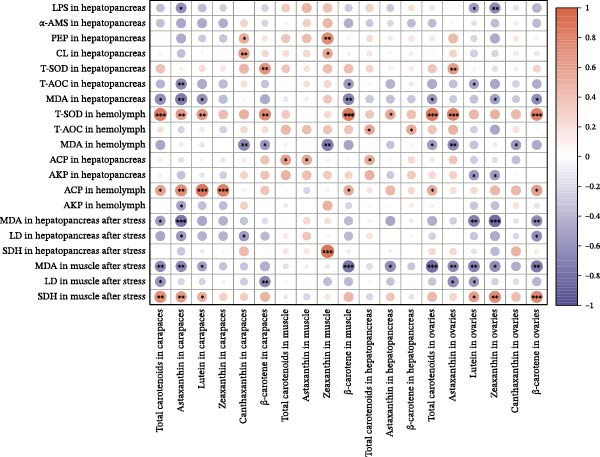
A heatmap illustrates the correlations between carotenoid levels in various tissues and the physiological indices of red swamp crayfish. Red bars are positive, purple bars are negative, and  ^∗^,  ^∗∗^, and  ^∗∗∗^ mark significance at *p*  < 0.05, *p*  < 0.01, and *p*  < 0.001, respectively.

As shown in Figure 9, the digestive enzymes in the hepatopancreas show relatively weak correlations with the carotenoid content in tissues. The T‐SOD activity in the hemolymph was highly significant and strongly positively correlated with the total carotenoids in the carapaces, the β‐carotene in the muscles, and the total carotenoids, astaxanthin, and β‐carotene in the ovaries (*p* < 0.001). The ACP activity in the hemolymph was also highly significant and positively correlated with the lutein and zeaxanthin in the carapaces (*p* < 0.001). After air exposure stress, the MDA content in the hepatopancreas was significantly negatively correlated with the astaxanthin in the carapaces and the lutein and zeaxanthin in the ovaries (*p* < 0.01). Additionally, the SDH activity in the muscles was significantly positively correlated with the astaxanthin in the carapaces and the zeaxanthin in the ovaries (*p* < 0.01). Tissue pigment accumulation reflected differences in AOC, immunity, and resistance to air exposure stress of crayfish.

## 4. Discussion

### 4.1. Growth Performance, Ovary Development, and Digestive Capacity

*D. salina* ranks among the richest natural β‐carotene producers [28]. In this study, although the highest WGR and SGR were observed in the 2.00% group, the lack of significant differences among treatments suggests that *D. salina* may not function primarily as a direct growth promoter. This aligns with findings in *P. monodon* [19], where β‐carotene supplementation improved health and pigmentation without consistently enhancing growth. The significant decline in growth performance in the D0.34 group, coupled with a markedly higher GSI compared to D0, D1.34, and D2.00 groups, suggests that 0.34% *D. salina* powder (48.01 mg kg^−1^ dry diet) significantly enhances ovarian development in red swamp crayfish, consistent with findings in red claw crayfish *C. quadricarinatus* [29]. We propose two nonexclusive mechanisms to explain this phenomenon: First, β‐carotene may directly protect oocytes from oxidative damage [30]. In crustaceans, both β‐carotene and astaxanthin steadily increase during ovarian maturation [31]. Deficiencies in carotenoids and retinol may lead to reduced reproductive capacity [32]. Second, within crustaceans, β‐carotene can be cleaved into retinal by β‐carotene oxygenase 1. Retinal is then reduced to retinol (vitamin A) by retinaldehyde reductase [33], a classic promoter of ovarian development in crustaceans [34, 35]. Recent studies indicate that vitamin A can promote vitellogenesis and subsequently advance ovarian development in *P. clarkii* by upregulating the mRNA expression of Vg and activating the ECR/RXR signaling pathway, while insufficient or excessive supplementation impedes ovarian maturation [36]. Higher *D. salina* inclusion levels reduced the stimulatory effect on gonad development, and more energy appeared to be allocated toward somatic growth, as evidenced by WGR.

*D. salina* has been demonstrated to play a significant role in promoting ovarian development and improving production performance in animals [21, 37]. The limited efficacy of high‐dose *D. salina* powder in promoting sustained ovarian development may be attributed to two potential mechanisms, though these require further validation. First, an appropriate amount of β‐carotene can be converted to astaxanthin, which is known to promote ovarian development [38, 39]. However, excessive intake may exceed metabolic capacity, and the accumulation of unused astaxanthin in the ovaries could potentially induce oxidative stress [3], thereby inhibiting the maturation process. Second, β‐carotene is metabolized to retinol [40]. An appropriate level of vitamin A promotes ovarian and reproductive development in crustaceans [41, 42], but excess may trigger lipid peroxidation, leading to increased MDA concentrations and hindering vitellogenin deposition [43]. Additionally, hepatopancreatic digestive enzyme activities increased initially and then declined with higher *D. salina* inclusion, peaking in the D0.34 group. These enzymatic changes correlated with elevated GSI, suggesting that 0.34% *D. salina* powder enhanced ovarian development by stimulating digestive enzyme activity, improving nutrient absorption, and activating retinol metabolism. This study reveals the potential promoting effect of low‐dose *D. salina* powder supplementation on ovarian development in *P. clarkii*, although the mechanisms involved remain hypothetical. Future studies employing transcriptomic or metabolomic approaches are necessary to directly track the metabolism of dietary β‐carotene from *D. salina* in red swamp crayfish, quantify intermediate products such as retinal and retinol, and elucidate the expression of key genes involved in carotenoid cleavage and retinoid signaling pathways.

### 4.2. Nonspecific Immunity, Antioxidant Status, and Air Exposure Stress Tolerance

In recent years, the declining immune performance of red swamp crayfish has increased their susceptibility to stress‐induced mortality, threatening the sustainability of the crayfish industry [44]. Algae, known for their rich content of bioactive compounds, play a crucial role in promoting animal health and immunity [45]. *D. salina* can be used as an immunostimulant, significantly improving the immune function of crustaceans. This effect has been experimentally confirmed in the shrimp *P. monodon* [46] and *L. vannamei* [47]. In this study, crayfish fed with a diet supplemented with 1.34% *D. salina* powder exhibited a significant increase in AKP activity in the hepatopancreas and ACP activity in the hemolymph, indicating enhanced immune status. Moreover, dietary β‐carotene derived from *D. salina* represents a practical strategy for improving AOC of aquatic animals [18], consistent with findings in the crab *E. sinensis* [17]. In this experiment, the addition of 0.67% *D. salina* powder resulted in a significant increase in T‐SOD activity and T‐AOC, coupled with markedly reduced hepatopancreatic and hemolymph MDA levels. However, excessive supplementation (1.34% and 2.00%) decreased T‐AOC in the hepatopancreas and an increased MDA in the hemolymph. Moderate supplementation with *D. salina* powder significantly enhanced AOC, primarily attributed to the activation of the antioxidant defense system by antioxidant components such as β‐carotene [17]. However, excessive intake of β‐carotene may inhibit the synthesis or activity of antioxidant enzymes. This could be due to sufficient antioxidant accumulation, leading to a feedback mechanism that downregulates endogenous antioxidant enzyme activity to maintain redox balance [48]. Alternatively, high‐dose supplementation may increase metabolic burden, thereby reducing antioxidant activity and immune function [22]. Therefore, appropriate supplementation with *D. salina* can significantly improve the immune function and AOC of red swamp crayfish, while excessive supplementation may diminish these benefits.

It is noteworthy that the limited tolerance of red swamp crayfish to transportation poses a significant constraint on the cross‐regional transfer of breeding stock and commercial market sales, hindering the sustainable expansion of crayfish farming. MDA serves as an indicator to evaluate the recovery from air exposure stress of red swamp crayfish [49]. In this study, the dietary supplementation of 2.00% *D. salina* powder significantly reduced MDA content in hepatopancreas and muscle tissues, an indication that oxidative stress imposed by air exposure was alleviated. Furthermore, muscle from the D2.00 group displayed substantially lower LD content and the highest SDH activity, reinforcing the conclusion that 2.00% *D. salina* supplementation can significantly enhance the air exposure tolerance and transportation resilience of crayfish. The intake of a high dosage of *D. salina* (2%) promoted greater deposition of carotenoids in the tissues, particularly astaxanthin in the carapaces and lutein/zeaxanthin in the ovaries (Figure 6), during stress (such as air exposure), the system may not rely solely on the endogenous antioxidant enzyme system. Instead, the antioxidants stored in the tissues may directly neutralize ROS, manifesting as higher stress tolerance. As reported in relevant studies on crustaceans, pigment accumulation may serve as a protective mechanism against adverse environmental conditions [50].

### 4.3. Correlation Between Carotenoid Content and Pigmentation Parameters, as Well as Physiological Indicators

Carotenoids from *D. salina*, which are known for high bioavailability, were effectively absorbed and utilized by red swamp crayfish, as evidenced by strong correlations between dietary levels and tissue accumulation in the carapaces and ovaries. The current result was consistent with the established pattern of carotenoid deposition observed in crustaceans [51, 52]. Among them, astaxanthin in the carapaces, lutein, and zeaxanthin in the ovaries all showed a linear accumulation trend in relation to dietary *D. salina* supplementation, while lutein in the carapaces and β‐carotene in the ovaries demonstrated a quadratic relationship, suggesting that there is a certain deposition threshold for carotenoids in the crayfish tissues. The dynamic changes in ovarian β‐carotene levels may explain the limited sustained promotion of ovarian development by *D. salina* supplementation. Crustacean pigmentation is closely linked to the absorption, transport, and metabolic transformation of carotenoids [53]. Dietary *D. salina* supplementation significantly increased the *a* ^∗^ value of the carapaces while decreasing the *L* ^∗^ and *b* ^∗^ values, indicating enhanced the external coloration driven by β‐carotene, likely via its metabolic conversion to astaxanthin. In this study, the *a* ^∗^, *L* ^∗^, and *b* ^∗^ color parameters of the carapaces correlated significantly with the total carotenoid content, consistent with findings in *E. sinensis* [54] and *Macrobrachium rosenbergii* [55]. Previous researches have shown that in the crab *E. sinensis*, the ovarian *a* ^∗^ value was positively correlated with astaxanthin content, whereas the *L* ^∗^ and *b* ^∗^ values declined linearly with rising astaxanthin and β‐carotene [56, 57]. Similarly, the ovarian *L* ^∗^ value also dropped sharply as astaxanthin increased. These results indicate that *D. salina* powder, when used as a feed additive for crayfish, has the potential to modulated carotenoid metabolism and thereby enhance marketable coloration cost‐effectively and sustainably.

Our findings corroborate the paradigm that optimal pigmentation is not merely a cosmetic trait but a tangible indicator of physiological health in farmed crustaceans [58]. The strong correlations between tissue carotenoid levels and key immune and antioxidant parameters (Figures 8 and 9) provide direct evidence for this link. This is attributed to the essential roles that carotenoids play in modulating the immune responses and antioxidant defense mechanisms [59]. In this study, hemolymph ACP activity tightly tracked carapace lutein and zeaxanthin contents, consistent with the positive relationship between carotenoid concentration and proPO system activity in *Gammarus pulex* [60]. Furthermore, T‐SOD activity in the hemolymph was significantly positively correlated with total carotenoids in the carapaces, β‐carotene in the muscles, and total carotenoids, astaxanthin, and β‐carotene in the ovaries. Similarly, Wei et al. [61] reported blue‐strain red‐claw crayfish, containing less astaxanthin and β‐carotene, displayed diminished SOD activity under high‐temperature stress relative to blue–green strain crayfish. These findings strongly suggest that carotenoids play a specific and significant role in augmenting the SOD‐mediated antioxidant defense system [62]. The precise pathways through which carotenoids modulate SOD activity; however, remain to be elucidated and represent a promising avenue for future research. In summary, the strong correlations between tissue carotenoid accumulation and key immune and antioxidant parameters indicate a close relationship between pigmentation and health status. Regression optimization pinpointed 1.23%–1.53% dietary *D. salina* as the range maximizing carapace total carotenoids and lutein, as well as ovarian total carotenoids and β‐carotene, significantly enhancing immune function and AOC of the crayfish, suggesting an optimal health condition. Furthermore, linear accumulation of pigments related to respiratory metabolic indicators demonstrated that the strongest resistance to air exposure was achieved at a *D. salina* supplementation level of 2.00%.

### 4.4. Industrial Implications

Natural astaxanthin is characterized by its high bioactivity but also high cost, which has driven extensive research focus toward the more cost‐effective synthetic astaxanthin [63]. Recent studies suggest that synthetic β‐carotene holds potential as a substitute for synthetic astaxanthin in some aquatic species. For instance, in *L. vannamei*, dietary supplementation with synthetic β‐carotene can achieve production performance comparable to that of synthetic astaxanthin [4]. In *P. monodon*, both also yield similar growth‐promoting effects [19]. Compared to synthetic β‐carotene, *D. salina* powder, as a premium natural source, is superior due to the comprehensive health benefits observed in this study, including enhanced growth, ovarian development, and improved stress resistance. Although this study did not include a direct comparison with synthetic pigments, the data indicate that *D. salina*‐derived β‐carotene can be efficiently converted to astaxanthin in crayfish tissues (Table 4), supporting its potential as an effective precursor from a metabolic perspective. More importantly, *D. salina* is a renewable and sustainable biological resource with relatively simple cultivation requirements, making it promising for commercial‐scale production.

This study provides guidance for the application of *D. salina* in crustacean aquaculture. First, we systematically quantified, for the first time, the dose–response relationship between *D. salina* supplementation and carotenoid deposition in the carapaces and ovaries of red swamp crayfish, establishing a precise optimal inclusion range (1.23%–1.53%) for improving coloration and health status. Second, this study revealed that a low dosage (0.34%) significantly promotes ovarian development, while higher dosages favor somatic growth. Notably, we are the first to demonstrate that *D. salina* can significantly enhance the tolerance of crayfish to air‐exposure stress. These findings offer practical and actionable strategies to support the transition of the crayfish industry toward high‐quality and sustainable production. A precision nutrition strategy based on natural and sustainable microalgal resources holds promise for enhancing the overall profitability of the red swamp crayfish farming industry.

## 5. Conclusion

This study shows that dietary *D. salina* powder provides dose‐dependent and multifaceted benefits in juvenile red swamp crayfish. Regression analysis identified an optimal inclusion range of 1.23%–1.53% for the concurrent enhancement of carotenoid deposition, immune function, and AOC, representing a holistic approach to improving crayfish health and market traits. Beyond this core optimum, specific inclusion levels were found to target distinct physiological outcomes: a lower dose of 0.34% preferentially stimulated ovarian development, whereas a higher dose of 2.00% maximized resilience to air exposure stress. Consequently, the strategic application of *D. salina* in functional feed formulations can be precisely calibrated to farming priorities, whether for superior product coloration, broodstock development, or transport survival. These data‐driven insights facilitate a strategic shift from quantity‐focused to quality‐ and value‐driven aquaculture practices, underpinning the sustainable and profitable development of the crayfish industry.

## Acknowledgments

The authors affirm that no AI tools were employed in the composition of this manuscript.

## Funding

This work was supported by the Special Fund of the Chinese Agriculture Research System from the Ministry of Agriculture of China (Grant CARS‐48), the key project of the NSFC‐Shandong Province Joint Fund from the Natural Science Foundation of China (Grant U1706209), and the Key R&D Program from the Ministry of Agriculture and Rural Affairs of China (Grant 2023YFD2402000).

## Disclosure

All contributors have examined the final draft and consented to its submission for publication.

## Ethics Statement

All experimental procedures were approved by the Shanghai Ocean University Animal Ethics Committee.

## Conflicts of Interest

The authors declare no conflicts of interest.

## Data Availability

The data that support the findings of this study are available from the corresponding author upon reasonable request.

## References

[1] Bureau of Fisheries and Management , China Fisheries Statistical Yearbook (2025), 2025, China Agriculture Press.

[2] China Fish , Development Report of Crayfish Industry in China, 2025, 2025, 7, National Fisheries Technology Extension Center, 10–16, (in Chinese).

[3] Zhang Y. , Qian C. , and Huang J. , et al.Suitable Natural Astaxanthin Supplementation With *Haematococcus pluvialis* Improves the Physiological Function and Stress Response to Air Exposure of Juvenile Red Swamp Crayfish (*Procambarus clarkii*), Aquaculture. (2023) 573, 10.1016/j.aquaculture.2023.739577, 739577.

[4] Fawzy S. , Wang W. L. , and Zhou Y. , et al.Can Dietary β-Carotene Supplementation Provide an Alternative to Astaxanthin on the Performance of Growth, Pigmentation, Biochemical, and Immuno-Physiological Parameters of *Litopenaeus vannamei* ?, Aquaculture Reports. (2022) 23, 10.1016/j.aqrep.2022.101054, 101054.

[5] Jiang X. , Zhu S. , Zhang G. , Gong Y. , and Wu X. , Effect of Dietary β-Carotene Supplementation on Growth and Antioxidant Capability of Pre–Adult Male Chinese Mitten Crab, *Eriocheir sinensis* , Aquaculture Reports. (2024) 35, 10.1016/j.aqrep.2024.101968, 101968.

[6] Ma T. , He J. , Jiang X. , and Hu Q. , Effect of Dietary β-Carotene Supplementation on Growth and Antioxidant Capability of Female Swimming Crab *Portunus trituberculatus* , Journal of the World Aquaculture Society. (2024) 55, no. 4, 10.1111/jwas.13073.

[7] Xue Y. , Wang Z. , Liu M. , Yi G. , Huang X. , and Wang W. , From Indicator Evaluation to Optimization Decision: Effects of Synthetic vs. Natural Astaxanthin on Pigmentation, Growth, and Health in *Penaeus vannamei* , Aquaculture. (2025) 609, 10.1016/j.aquaculture.2025.742782, 742782.

[8] Abdel-Latif H. M. R. , El-Ashram S. , and Sayed A. E.-D. H. , et al.Elucidating the Ameliorative Effects of the Cyanobacterium Spirulina (*Arthrospira platensis*) and Several Microalgal Species Against the Negative Impacts of Contaminants in Freshwater Fish: A Review, Aquaculture. (2022) 554, 10.1016/j.aquaculture.2022.738155, 738155.

[9] Wen X. , Chen J. , and Liu C. , et al.Potential of a Novel Marine Microalgae *Dunaliella* sp. MASCC-0014 as Feed Supplements for Sustainable Aquaculture, Aquaculture. (2025) 596, no. 1, 10.1016/j.aquaculture.2024.741732, 741732.

[10] Rodriguez-Amaya D. B. , Esquivel P. , and Meléndez-Martínez A. J. , Comprehensive Update on Carotenoid Colorants From Plants and Microalgae: Challenges and Advances From Research Laboratories to Industry, Foods. (2023) 12, no. 22, 10.3390/foods12224080, 4080. 38002140 PMC10670565

[11] Šimat V. , Rathod N. , Čagalj M. , Hamed I. , and Generalić Mekinić I. , Astaxanthin From Crustaceans and Their Byproducts: A Bioactive Metabolite Candidate for Therapeutic Application, Marine Drugs. (2022) 20, no. 3, 10.3390/md20030206, 206. 35323505 PMC8955251

[12] Liu C. , Liu H. , and Zhu X. , et al.The Effects of *Arthrospira platensis* and Lutein on the Growth, Antioxidant Capacity and Pigmentation in Hybrid Yellow Catfish (*Pelteobagrus fulvidraco* ♀ × *Pelteobagrus vachelli* ♂), Acta Hydrobiologica Sinica. (2021) 45, 1024–1033, (in Chinese with English abstract) 10.7541/2021.2020.139.

[13] Lou G. , Guo Y. , and Liu X. , et al.Dietary Synthetic Astaxanthin and Natural Astaxanthin From *Haematococcus pluvialis* and *Phaffia rhodozyma* Improves the Growth, Antioxidant Capacity, Innate Immunity, and Pigmentation of Pacific White Shrimp (*Litopenaeus vannamei*), Aquaculture Nutrition. (2025) 2025, no. 1, 10.1155/anu/8822600, 8822600. 41031291 PMC12479155

[14] Aditi , Bhardwaj R. , Yadav A. , Swapnil P. , and Meena M. , Characterization of Microalgal β-Carotene and Astaxanthin: Exploring Their Health–Promoting Properties Under the Effect of Salinity and Light Intensity, Biotechnology for Biofuels and Bioproducts. (2025) 18, no. 1, 10.1186/s13068-025-02612-x. PMC11829443 39953577

[15] de Souza Celente G. , de Cassia de Souza Schneider R. , Medianeira Rizzetti T. , Lobo E. A. , and Sui Y. , Using Wastewater as a Cultivation Alternative for Microalga *Dunaliella salina*: Potentials and Challenges, Science of the Total Environment. (2024) 911, 10.1016/j.scitotenv.2023.168812, 168812. 38000734

[16] Huang B. , Qu G. , He Y. , Zhang J. , Fan J. , and Tang T. , Study on High–CO_2_ Tolerant *Dunaliella salina* and Its Mechanism via Transcriptomic Analysis, Frontiers in Bioengineering and Biotechnology. (2022) 10, 10.3389/fbioe.2022.1086357, 1086357. 36532596 PMC9751823

[17] Liu M. , Jiang X. , Chen A. , Chen T. , Cheng Y. , and Wu X. , Transcriptome Analysis Reveals the Potential Mechanism of Dietary Carotenoids Improving Antioxidative Capability and Immunity of Juvenile Chinese Mitten Crabs *Eriocheir sinensis* , Fish & Shellfish Immunology. (2020) 104, 359–373, 10.1016/j.fsi.2020.06.033. 32553983

[18] Félix D. M. , Jacinto E. C. , and Campa Córdova Á.I. , et al.Physiological and Antioxidant Response of *Litopenaeus vannamei* Against *Vibrio parahaemolyticus* Infection After Feeding Supplemented Diets Containing *Dunaliella* sp. Flour and β-Glucans, Journal of Invertebrate Pathology. (2022) 187, 10.1016/j.jip.2021.107702, 107702. 34902396

[19] Boonyaratpalin M. , Thongrod S. , Supamattaya K. , Britton G. , and Schlipalius L. E. , Effects of β-Carotene Source, *Dunaliella salina*, and Astaxanthin on Pigmentation, Growth, Survival and Health of *Penaeus monodon* , Aquaculture Research. (2001) 32, no. 1, 182–190, 10.1046/j.1355-557x.2001.00039.x, 2-s2.0-23044438828.

[20] Harpaz S. , Rise M. , Arad S. M. , and Gur N. , The Effect of Three Carotenoid Sources on Growth and Pigmentation of Juvenile Freshwater Crayfish *Cherax quadricarinatus* , Aquaculture Nutrition. (1998) 4, no. 3, 201–208, 10.1046/j.1365-2095.1998.00067.x, 2-s2.0-0242722311.

[21] Senosy W. , Kassab A. Y. , and Mohammed A. A. , Effects of Feeding Green Microalgae on Ovarian Activity, Reproductive Hormones and Metabolic Parameters of Boer Goats in Arid Subtropics, Theriogenology. (2017) 96, 16–22, 10.1016/j.theriogenology.2017.03.019, 2-s2.0-85016438085. 28532834

[22] Akbari P. , Gholamhosseini A. , Ali M. , Aminikhoei Z. , Tavabe K. R. , and Kuchaksaraei B. S. , Growth, Fatty Acid Composition, Antioxidant Activity and Resistance of *Litopenaeus vannamei* Fed With *Dunaliella salina* , Iranian Journal of Science. (2023) 47, no. 1, 35–45, 10.1007/s40995-022-01396-1.

[23] AOAC , Official Methods of Analysis of AOAC International, 2006, eighteenth edition, Association of Official Analytical Chemists.

[24] Folch J. , Lees M. , and Stanley G. H. S. , A Simple Method for the Isolation and Purification of Total Lipides From Animal Tissues, Journal of Biological Chemistry. (1957) 226, no. 1, 497–509, 10.1016/S0021-9258(18)64849-5. 13428781

[25] Cheng Z. , Shi J. , and Qian C. , et al.The Enhanced Growth Performance and Antioxidant Capacity of Juvenile *Procambarus clarkii* Fed With Microbial Antioxidants, Antioxidants. (2025) 14, no. 2, 10.3390/antiox14020135, 135. 40002322 PMC11851763

[26] CIE (International Commission on Illumination) , Colorimetry, 1976, 2nd edition, Central Bureau of the CIE.

[27] Long X. , Wu X. , Zhao L. , Liu J. , and Cheng Y. , Effects of Dietary Supplementation With *Haematococcus pluvialis* Cell Powder on Coloration, Ovarian Development and Antioxidation Capacity of Adult Female Chinese Mitten Crab, *Eriocheir sinensis* , Aquaculture. (2017) 473, 545–553, 10.1016/j.aquaculture.2017.03.010, 2-s2.0-85015440199.

[28] Pérez-Legaspi I. A. , Valadez-Rocha V. , Ortega-Clemente L. A. , and Jiménez-García M. I. , Microalgal Pigment Induction and Transfer in Aquaculture, Reviews in Aquaculture. (2020) 12, no. 3, 1323–1343, 10.1111/raq.12384, 2-s2.0-85073948473.

[29] Linan-Cabello M. A. , Medina-Zendejas R. , Sanchez-Barajas M. , and Mena Herrera A. , Effects of Carotenoids and Retinol in Oocyte Maturation of Crayfish *Cherax quadrucarinatus* , Aquaculture Research. (2004) 35, no. 9, 905–911, 10.1111/j.1365-2109.2004.01083.x, 2-s2.0-17144444677.

[30] Barim-Oz O. and Sahin H. , The Influence of Dietary Antioxidant on Ovarian Eggs and Levels of Vitamin E, C, A, Astaxanthin, β-Carotene and Oxidative Stress in Tissues of *Astacus leptodactylus* (Eschscholtz) During Reproduction, Cellular and Molecular Biology. (2017) 62, no. 14, 1–10, 10.14715/cmb/2016.62.14.1, 2-s2.0-85013092881. 28145851

[31] Jothy T. P. , Kannan R. R. , and Subramoniam T. , Hepatopancreatic Contributions of Lipids and Carotenoids to Vitellogenesis in the Intertidal Anomuran Crab, *Emerita asiatica* (Milne Edwards), Indian Journal of Experimental Biology. (2022) 60, 573–579, 10.56042/ijeb.v60i08.34564.

[32] Liñán-Cabello M. A. , Paniagua-Michel J. , and Zenteno-Savín T. , Carotenoids and Retinal Levels in Captive and Wild Shrimp, *Litopenaeus vannamei* , Aquaculture Nutrition. (2003) 9, no. 6, 383–389, 10.1046/j.1365-2095.2003.00267.x, 2-s2.0-2442487905.

[33] Liao Y. , Zhang B. , and Wang D. , et al.Metabolism, Function, Molecular Mechanism, and Application of Carotenoids in Coloration of Aquatic Animals, Reviews in Aquaculture. (2025) 17, no. 2, 10.1111/raq.70016.

[34] Liñán-Cabello M. A. , Paniagua-Michel J. , and Hopkins P. M. , Bioactive Roles of Carotenoids and Retinoids in Crustaceans: Carotenoids and Retinoids in Crustaceans, Aquaculture Nutrition. (2002) 8, no. 4, 299–309, 10.1046/j.1365-2095.2002.00221.x, 2-s2.0-19044361824.

[35] Liñán-Cabello M. A. and Paniagua-Michel J. , Induction Factors Derived From Carotenoids and Vitamin A During the Ovarian Maturation of *Litopenaeus vannamei* , Aquaculture International. (2004) 12, no. 6, 583–592, 10.1007/s10499-004-1088-7.

[36] Yu J. , Hu S. , and Zhang F. , et al.Nutritional Regulation by Vitamin A: Comprehensive Impacts on Growth, Molting, Immunity, Lipid Metabolism, and Ovarian Maturation in Red Swamp Crayfish (*Procambarus clarkii*), Fish & Shellfish Immunology. (2026) 168, 10.1016/j.fsi.2025.110948, 110948. 41106711

[37] Madkour M. , Ali S. I. , and Alagawany M. , et al.Dietary *Dunaliella salina* Microalgae Enriches Eggs With Carotenoids and Long-Chain Omega-3 Fatty Acids, Enhancing the Antioxidant and Immune Responses in Heat–Stressed Laying Hens, Frontiers in Veterinary Science. (2025) 12, 10.3389/fvets.2025.1545433, 1545433. 40078214 PMC11897048

[38] Pangantihon-Kühlmann M. P. , Millamena O. , and Chern Y. , Effect of Dietary Astaxanthin and Vitamin A on the Reproductive Performance of *Penaeus mondon* Broodstock, Aquatic Living Resources. (1998) 11, no. 6, 403–409, 10.1016/S0990-7440(99)80006-0, 2-s2.0-0032215662.

[39] Chen X. , Chen J. , and Huang L. , et al. *PcASTA* in *Procambarus clarkii*, a Novel Astaxanthin Gene Affecting Shell Color, Frontiers in Marine Science. (2024) 10, 10.3389/fmars.2023.1343126, 1343126.

[40] Zhang X. L. , Wang G. D. , Huang S. Y. , Gong X. T. , and Wang Y. L. , Research Progress on the Mechanism of Carotenoid Absorption, Metabolism and Deposition in Animals in Aquature: A Review, Journal of Dalian Ocean University. (2023) 38, no. 6, 1072–1082, (in Chinese with English abstract) 10.16535/j.cnki.dlhyxb.2023-053.

[41] Mengqing L. , Wenjuan J. , Qing C. , and Jialin W. , The Effect of Vitamin A Supplementation in Broodstock Feed on Reproductive Performance and Larval Quality in *Penaeus chinensis* , Aquaculture Nutrition. (2004) 10, no. 5, 295–300, 10.1111/j.1365-2095.2004.00302.x, 2-s2.0-6344277470.

[42] Huang Q. , Wang X. , and Bu X. , et al.Role of Vitamin A in the Ovary Development for Female *Eriocheir sinensis* in the Gonadal Development Stage, Aquaculture. (2022) 560, 10.1016/j.aquaculture.2022.738612, 738612.

[43] Huang Q.-C. , Wang L. , and Gu Z.-M. , et al.Dietary Retinoic Acid Improved the Growth, Lipid Metabolism and Immune Status in *Macrobrachium rosenbergii* , Frontiers in Marine Science. (2025) 12, 10.3389/fmars.2025.1567872, 1567872.

[44] Ren X. , Peng G. , Peng B. , Tan Y. , and Bai X. , Robust Strategy for Disease Resistance and Increasing Production Breeding in Red Swamp Crayfish (*Procambarus clarkii*), Fish & Shellfish Immunology. (2022) 122, 57–66, 10.1016/j.fsi.2022.01.032. 35085739

[45] Saddiqa A. , Faisal Z. , and Akram N. , et al.Algal Pigments: Therapeutic Potential and Food Applications, Food Science & Nutrition. (2024) 12, no. 10, 6956–6969, 10.1002/fsn3.4370. 39479711 PMC11521690

[46] Li Y. , Xiao G. , Mangott A. , Kent M. , and Pirozzi I. , Nutrient Efficacy of Microalgae as Aquafeed Additives for the Adult Black Tiger Prawn, *Penaeus monodon* , Aquaculture Research. (2016) 47, no. 11, 3625–3635, 10.1111/are.12815, 2-s2.0-84932143787.

[47] Medina-Félix D. , López-Elías J. A. , and Martínez-Córdova L. R. , et al.Evaluation of the Productive and Physiological Responses of *Litopenaeus vannamei* Infected With WSSV and Fed Diets Enriched With *Dunaliella* sp, Journal of Invertebrate Pathology. (2014) 117, 9–12, 10.1016/j.jip.2013.12.004, 2-s2.0-84896515560. 24424376

[48] Zhang J. , Liu Y. J. , and Tian L. X. , et al.Effects of Dietary Astaxanthin on Growth, Antioxidant Capacity and Gene Expression in Pacific White Shrimp *Litopenaeus vannamei* , Aquaculture Nutrition. (2013) 19, no. 6, 917–927, 10.1111/anu.12037, 2-s2.0-84887621543.

[49] Lei X. , Yang L. , and Tan L. , et al.Effect of Air Exposure and Re–Submersion on the Histological Structure, Antioxidant Response, and Gene Expression of *Procambarus clarkii* , Animals. (2023) 13, no. 3, 10.3390/ani13030462, 462. 36766351 PMC9913771

[50] Babin A. , Moreau J. , and Moret Y. , Storage of Carotenoids in Crustaceans as an Adaptation to Modulate Immunopathology and Optimize Immunological and Life–History Strategies, BioEssays. (2019) 41, no. 11, 10.1002/bies.201800254, 2-s2.0-85074184682, 1800254. 31566782

[51] Castillo R. and Nègre-Sadargues G. , Effect of Different Dietary Carotenoids on the Pigmented Pattern of the Hermit Crab, *Clibanarius erythropus*, Latreille (Crustacea: Decapoda), Comparative Biochemistry and Physiology Part A: Physiology. (1995) 111, no. 4, 533–538, 10.1016/0300-9629(95)00074-H, 2-s2.0-0029075918.

[52] Díaz A. C. , Velurtas S. M. , Fernández Gimenez A. V. , Mendiara S. N. , and Fenucci J. L. , Carotenoids in Integument, Muscle, and Midgut Gland of Red Shrimp, *Pleoticus muelleri*, Bate, 1888 (Crustacea, Penaeoidea) Fed Carotenoid–Supplemented Diets, Israeli Journal of Aquaculture–Bamidgeh. (2011) 63, 625.

[53] Huang Y. , Zhang L. , Wang G. , and Huang S. , De Novo Assembly Transcriptome Analysis Reveals the Genes Associated With Body Color Formation in the Freshwater Ornamental Shrimps *Neocaridina denticulate sinensis* , Gene. (2022) 806, 10.1016/j.gene.2021.145929, 145929. 34461150

[54] Li Q. , Sun Q. , Liu Q. , Cheng Y. , and Wu X. , Estimation of Genetic Parameters for Carotenoid Traits in Chinese Mitten Crab, *Eriocheir sinensis*, Females, Aquaculture. (2021) 532, 10.1016/j.aquaculture.2020.735990, 735990.

[55] Li Q. , Liao J. , and Lin L. , The-Raw Shell Color Determines Cooked Color and Carotenoid Profiles of the Freshwater Prawn *Macrobrachium rosenbergii* (De Man, 1879) (Decapoda: Caridea: Palaemonidae), Journal of Crustacean Biology. (2023) 43, no. 1, 1–7, 10.1093/jcbiol/ruad013.

[56] Zhang R. , Zhang L. , Jiang X. , Wu X. , and Wang X. , Effects of Dietary β-Carotene on Color and Flavor Quality of Ovaries in Adult Female Chinese Mitten Crab (*Eriocheir sinensis*), 2024, 5, no. 4, 10.1002/efd2.169.

[57] Zhang L. , Zhang R. , Jiang X. , Wu X. , and Wang X. , Dietary Supplementation With Synthetic Astaxanthin and DHA Interactively Regulates Physiological Metabolism to Improve the Color and Odor Quality of Ovaries in Adult Female *Eriocheir sinensis* , Food Chemistry. (2024) 430, 10.1016/j.foodchem.2023.137020, 137020. 37544156

[58] Alfakih A. , Watt P. J. , and Nadeau N. J. , The Physiological Cost of Colour Change: Evidence, Implications and Mitigations, Journal of Experimental Biology. (2022) 225, no. 10, 10.1242/jeb.210401, 210401. 35593398

[59] Babin A. , Saciat C. , and Teixeira M. , et al.Limiting Immunopathology: Interaction Between Carotenoids and Enzymatic Antioxidant Defences, Developmental and Comparative Immunology. (2015) 49, no. 2, 278–281, 10.1016/j.dci.2014.12.007, 2-s2.0-84937120271. 25524820

[60] Cornet S. , Biard C. , and Moret Y. , Is There a Role for Antioxidant Carotenoids in Limiting Self–Harming Immune Response in Invertebrates?, Biology Letters. (2007) 3, no. 3, 284–288, 10.1098/rsbl.2007.0003, 2-s2.0-38449102049. 17374587 PMC2464685

[61] Wei M. , Wang A. M. , and Gu Z. F. , et al.Comparison of Growth Performance, Carotenoid Content, and Temperature Tolerance of Two–Colored Strains of the Red Claw Crayfish *Cherax quadricarinatus* , Journal of Shellfish Research. (2021) 40, no. 2, 421–427, 10.2983/035.040.0214.

[62] Deng Y. , Zhan W. , and Xie S. , et al.Multi–Omics Analysis Revealed the Effects of Different Astaxanthin Sources on the Antioxidant Properties of *Scylla paramamosain* , Food Chemistry. (2025) 478, 10.1016/j.foodchem.2025.143470, 143470. 40049124

[63] Chen Q. , Huang S. , and Dai J. , et al.Effects of Synthetic Astaxanthin on the Growth Performance, Pigmentation, Antioxidant Capacity, and Immune Response in Black Tiger Prawn (*Penaeus monodon*), Aquaculture Nutrition. (2023) 2023, 10.1155/2023/6632067, 6632067. 38161983 PMC10756741

